# Effectiveness of an e-Health Quasi-Randomized Controlled Universal Prevention Program for Eating Disorders in Spanish Adolescents

**DOI:** 10.1007/s10935-023-00751-1

**Published:** 2023-10-31

**Authors:** Jorge Pérez-Vázquez, Alba González-Roz, Isaac Amigo-Vázquez

**Affiliations:** Department of Psychology, Faculty of Psychology, Plaza Feijoo S/N, 33003 Oviedo, Asturias Spain

**Keywords:** Eating disorders, e-Health, Adolescents, Internet-based intervention, Prevention

## Abstract

Eating disorders (EDs) and sub-threshold conditions are prevalent in the adolescent population. Unfortunately, most preventive interventions have been targeted at emerging adults and the effectiveness of online prevention programs has yet to be determined in adolescents. This study sought to examine the short-term effectiveness of a universal e-Health psychoeducational prevention program for EDs compared to a control (non-intervention) group in Spanish adolescents. Using a quasi-randomized trial design, a total of 161 [% girls: 45.96; Mage(SD) = 12.43 (0.43)] adolescents from 5 participating schools were allocated to two intervention arms: (1) psychoeducational intervention (n = 79) and (2) wait-list control (n = 82). The intervention was delivered over 3 months through 3 modules that were accessible 24/7 and 3 school sessions guided by the students´ tutors focusing on nutrition, promoting a healthy lifestyle, mitigating body concerns, and social pressures. Participants completed an online assessment battery including the Eating Attitudes Test (EAT-26) and measures of self-esteem, family disruption, compliance with the Mediterranean diet, and lifestyle. Correlational analysis showed small to moderate relationships between self-esteem and family function (*rho* = 0.413, *p* = 0.001), BMI (body mass index) and the EAT-26 dieting subscale (*rho* = 0.417, *p* = 0.001), physical activity and the bulimia subscale (*rho* =  − 0.237, *p* = 0.003), and self-esteem and the dieting subscale (*rho* =  − 0.223, *p* = 0.004). At the post-intervention assessment, the intervention group showed a statistically significant reduction in ED risk (EAT-26) (*d* =  − 0.323, *p* = 0.040) and the oral control subscale (*d* = 0.327, *p* = 0.038). The e-health intervention including tutor-led digital components was effective for reducing ED risk in children. Results must be interpreted with caution due to the low statistical power and the limited sample size. Large scale randomized controlled trials with longer follow-ups will be needed to bolster the evidence.

## Introduction

Adolescents and young adults are at high risk of developing an eating disorder (ED), and it is estimated that 55.2% of cases of anorexia nervosa (AN), 45.3% of cases of bulimia nervosa (BN) and 34.5% of cases of binge-eating disorder (BED) occur before the age of 18 years-old (Solmi et al., 2021). The highest incidence for AN are in girls and women aged 15–19, a group which makes up at least 40% of all identified cases (Hoek & Van Hoeken, 2003; Kusters et al., 2021). AN and BN peak age is close to mid-adolescence (15.5 years-old) and BED close to early-adulthood (19.5 years-old) in both sexes (Solmi et al., 2021).

Lifetime prevalence of EDs in the general population is 1.69%. Specifically by diagnostic category, the rate of AN is 0.16%, for BN it is 0.63% and for BED it is 0.16%. In Western countries, lifetime prevalence of any ED is greater than 1.89%, with increased percentages specially in women and girls of 2.58% (Qian et al., 2021). For instance, ED prevalence is 0.71% for Spanish boys and 3.64% for Spanish girls (Kusters et al., 2021). In adolescent girls in the US and Europe, lifetime ED prevalence rates are estimated at 0.3% for AN, 0.9–1% for BN, and 1–1.6% for BED (Hoek & Van Hoeken, 2003; Swanson et al., 2011). In Spain, the situation appears to be similar in terms of ED prevalence in 10–15 year-olds for AN and BN (0.4% in boys—0.8% in girls) and for OSFED (other specified feeding or eating disorders) (1.8% in boys, 1.9% in girls) (Subdirección General de Información Sanitaria, 2021).

Traditionally, in the field of EDs, much research has been devoted to selective prevention and treatment rather than to universal prevention. Risk factors that are typically addressed in both universal prevention and treatment include body self-esteem, dietary restraint, thin-ideal internalization and body dissatisfaction (i.e., media literacy) (Levine & Piran, 2004; Schwartz et al., 2019). Unlike treatment approaches that are particularly focused on restructuring weight-related negative distortions and co-occurrent conditions (e.g., anxiety, depression), universal prevention specifically aims to provide children with the abilities and skills that may deter them from internalizing the media propagated thin-ideal. In this capacity, interventions are aimed at critically appraising mass media publicity and providing education on body diversity along with promoting healthy lifestyles and eating habits (Paxton & McVey, 2012; Sánchez-Carracedo et al., 2017; Sánchez-Carracedo et al., 2012).

Recent years have seen growing interest in examining the effectiveness of traditional universal preventive interventions targeting ED in adolescents. Some studies indicated that only media literacy programs were effective in reducing shape and weight concerns at post-test and at follow-up (6–30 months) in boys and girls (Le et al., 2017). Media literacy intervention programs are linked not only to a reduction in negative body image (Kurz et al., 2021), but also to a decrease in eating concerns and thin-internalization attitudes (Zuair & Sopory, 2022). Other universal interventions for adolescents such as multicomponent based and self-esteem enhancement programs seem to be particularly effective only in girls (Le et al., 2017).

E-Health interventions for preventing EDs have been increasingly recognized (Aardoom et al., 2013; Melioli et al., 2016). At all ages, e-Health has been related to reduced concerns about shape and weight, ED symptomatology, dietary restraint, a drive for thinness and thin-ideal internalization (Linardon et al., 2020). Similarly, in adolescents and college students, e-Health interventions have produced significant reductions in ED symptoms (Harrer et al., 2019).

So far, e-Health interventions in adolescents have been tested in very few studies, showing small effects. Media literacy and advocacy skills programs produced a small improvement in the internalization of societal ideals relating to appearance at follow-up (3–5 months) (Kusel, 1999), and CBT e-Health interventions in overweight adolescents with binge eating episodes seem to be associated with a reduction in concerns about weight and shape, binge episodes, and BMI at follow-up (Jones et al., 2008).

Despite these valuable efforts, there is still much uncertainty about the effectiveness of universal e-Health preventive programs in adolescents, as most of the available studies have mainly focused on treatment approaches (Aardoom et al., 2013; Dölemeyer et al., 2013) or young adults (Bauer et al., 2009; Beintner et al., 2012).

Against this background, this study aimed to develop a universal, school based, psychoeducational e-Health preventive intervention for ED involving teachers and parents and examine its effectiveness. Specifically looking at its effectiveness in: (1) reducing risk of ED; (2) minimizing extreme BMI changes; (3) reducing shape concerns and drive-for-thinness attitudes; (4) reducing food preoccupation and bulimia-related behaviors; (5) reducing self-control behaviors and social pressure related to food; (6) enhancing adherence to the Mediterranean diet; (7) promoting an active lifestyle; (8) improving self-esteem; and (9) bolstering family functioning.

## Methods

### Participants and Procedure

All the procedures used in this study were in conformance with the Declaration of Helsinki and written informed consent from participants was collected before the beginning of the study. Approval was obtained from the ethics committee of the Principality of Asturias (n°123/18).

All participants belonged to one of the eight Health Areas in the Principality of Asturias in the north of Spain. Asturias has a total population of 1,022,800, a crude birth rate of 5.05 (births/1000 population), a crude death rate of 12.64 (deaths/1000 population), a life expectancy in both sexes of 82.62 years, a percentage of foreign people of 4.07%, a middle aged population of 48.33 years-old, and an aging index of 235.93% (% of people older than 64/younger than 15). The health area covers 59,462 inhabitants of three municipalities (Mieres, Aller and Lena). Specifically, in the 10–14 year-old age range, there were 1,907 adolescents in 2019. Of these adolescents, 49.74% (950) were girls, 96.54% (1844) were Spanish and 3.46% (66) were foreigners (Sociedad Asturiana de Estudios Económicos e Industriales, 2022).

The recruitment process took place between October and November 2019 in high schools within the VII Health Area of the Health Service of the Principality of Asturias (Spain). The heads of participating schools were contacted by mail or phone and face-to-face meetings were scheduled with children’s tutors to inform them of the study aims and procedures, and the need for informed consent.

Five out of the six state secondary schools in the area (83.33%) agreed to participate. Neither of the two subsidized secondary schools chose to participate. There are no private schools in the Health Area where the study was conducted.

The eligibility criteria for participating in this study were: (1) being aged 10–14 years old and (2) being in the first year of high school in the VII Health Area of the Health Service of the Principality of Asturias (Spain). Children were not included in the study if: (1) informed consent was not obtained from the children and their parents or legal guardians; (2) they had an intellectual disability or were not fluent in Spanish; (3) they were receiving psychiatric, psychological or nutritional treatment at the time of the study.

**Fig. 1:**
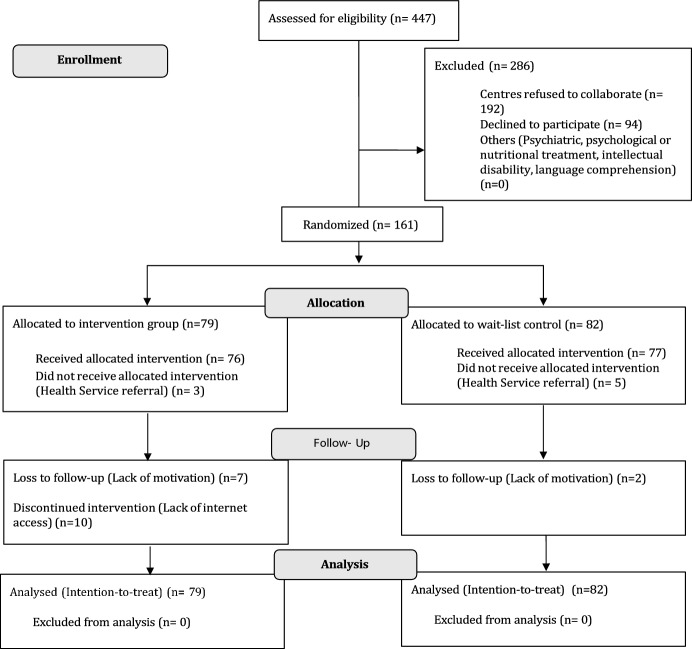
Flow diagram of the study participants

The participant flowchart (see Fig. 1) displays a detailed description of the number of participants through each stage of the study (i.e., enrollment, allocation and follow up). A total of 447 children were initially assessed. Of these, 286 (63.98%) were excluded (see detail in Fig. 1). Teachers delivered the prevention program only to children who had parental consent to participate. None of the excluded students were given access to the online content.

Following a quasi-randomized design, three of the five state schools that agreed to participate were allocated to the intervention group while the remaining two made up the control group. The final sample comprised 161 participants allocated to two arms: 1) an e-Health psychoeducational intervention (intervention group; IG) (n = 79) and 2) a wait-list control (WLC) (n = 82). The intervention was delivered between December 2019 and March 2020.

Parents of children with a score over 20 in the EAT-26 and a weight percentile indicating overweight, obesity or underweight at baseline met in a 30 min informative group session led by the researchers. During this session, they were given information and training in how to identify early signs of EDs. At the end of the session, participants at risk of EDs (i.e., EAT-26 > 20 and a weight percentile indicating overweight = above 85th, obesity = above 97th or underweight = below 3rd) were referred to their primary health care center for a more comprehensive evaluation. Parents/tutors were given written reports with the EAT-26 scores and weight percentiles.

Table 1 gives the baseline demographic characteristics by group, split by sex. The IG and WLC groups differed in weight, BMI, family function and physical activity, but the statistical power level associated with the statistical analyses were below 80%. There were statistically significant differences by sex in physical activity, but the statistical power was again below 80%. The two study groups did not differ in ED risk [*χ*^2^(1,*n* = 161) = 0.031, *p* = 0.861]. There were 17 subjects above the cut-off point of 20 in the EAT-26 test (Eating Attitudes Test) at the baseline assessment, 8 of whom were referred to the Health Service due to their percentile scores for overweight (n = 2), obesity (n = 6) or underweight (n = 0).

**Table 1:** Demographic characteristics

	Group					Sex				
Variable	IG = 79	WLC = 82	*p*	Effect	1 − β	Girls = 74	Boys = 87	*p*	Effect	1 − β
Girls (*n*)	37	37								
Boys (*n*)	42	45								
Age (*SD*)	12.46 (0.48)	12.51 (0.48)	0.409			12.43 (0.43)	12.54 (0.51)	0.251		
Weight (*SD*)	53.59 (12.71)	48.62 (11.56)	0.010*	0.410	0.498	50.52 (10.87)	51.52 (13.54)	0.513		
Height (*SD*)	154.20 (7.65)	153.00 (8.11)	0.336			153.25 (7.47)	153.87 (8.25)	0.499		
BMI (*SD*)	22.40 (4.40)	20.60 (3.86)	0.011*	0.401	0.489	21.38 (3.75)	21.57 (4.59)	0.863		
EAT-26 (*SD*)	9.44 (6.06)	9.20 (6.81)	0.472			8.70 (6.01)	9.84 (6.76)	0.345		
Sub.Dieting (*SD*)	4.70 (4.57)	4.72 (5.08)	0.747			3.96 (4.21)	5.36 (5.22)	0.130		
Sub.Bulimia (*SD*)	1.03 (2.25)	0.50 (0.93)	0.421			0.97 (2.20)	0.57 (1.16)	0.456		
Sub.oral (*SD*)	3.71 (2.92)	3.98 (3.07)	0.564			3.77 (3.28)	3.91 (2.73)	0.472		
RS (*SD*)	33.94 (5.19)	33.27 (4.70)	0.776			33.34 (5.23)	32.91 (4.68)	0.468		
Apgar (*SD*)	8.88 (1.52)	8.49 (1.58)	0.044*	0.323	0.477	8.78 (1.53)	8.60 (1.58)	0.384		
P.Activity (*SD*)	5.47 (2.20)	6.64 (2.01)	0.001*	0.519	0.444	5.49 (2.25)	6.56 (2.01)	0.003*	0.468	0.324
Nutrition (*SD*)	9.99 (2.72)	9.91 (2.80)	0.873			9.97 (2.85)	9.93 (2.69)	0.737		

*IG* Intervention group, *WLC* Wait-list control, *BMI* Body mass index, *EAT-26* Eating Attitudes Test, *Sub*.*Dieting* Dieting subscale, *Sub*.*Bulimia* Bulimia and food preoccupation subscale, *Sub*.*Oral* Oral control subscale, *RS* Rosenberg Self-esteem Scale, *Apgar* Family Apgar Test, *P*.*Activity* Short Test (Physical Activity), *Nutrition* Quick Test (Nutrition)

*Statistically significant *p* < 0.05

### Assessment Measures

Members of the research team visited the schools before and after the intervention and asked participants to provide information on sociodemographic, anthropometric, eating-related, psychological, nutritional and lifestyle measures.

Besides the aforementioned variables, the battery assessment included a total of 7 attention control items to detect inconsistent data and lack of attention/effort in multiple choice questionnaires (e.g. “For this question please choose completely agree”). To link pre-post data, a randomly generated 6-digit numerical code in the platform was assigned to each of the participants.

We used Moodle^®^ to collect self-reported measures at baseline and post-intervention. Each participant received a personal user ID (consisting of an alphanumeric ID) and a password to access Moodle^®^. All pre-intervention data were collected in a single assessment session that took 50 min between December 2019 and January 2020. The post-intervention assessment took a similar length of time and was conducted in April 2020.

#### Sociodemographic and Anthropometric Measures

Participants provided information on sociodemographic characteristics (i.e., date of birth, sex, country of birth, ethnicity, household size, parents’ educational attainment, etc.).

Weight and height were collected by researchers, BMI and individual’s weight percentiles according to age were calculated. Values below the 3rd percentile were considered underweight, values above the 85th percentile were considered overweight, and values above the 97th percentile were considered obesity (Serra Majem et al., 2003).

#### Eating-Related Measures

The EAT-26 (Garner et al., 1982), a 26-item self-reported questionnaire, was used to assess ED risk. Items are presented on a 6-point Likert scale with responses ranging from 1 “never” to 6 “always”. The total score ranges from 0 to 78. A score of 20 indicates a high risk of ED (high level of concern about dieting, body weight or problematic eating behaviors) and suggests the need to conduct a deeper psychological assessment. The EAT-26 has three subscales: (1) dieting, (2) bulimia and food preoccupation, and (3) oral control.

#### Psychological Measures

The Family Apgar Test adapted for children (Austin & Huberty, 1989), was used to assess family members´ satisfaction with each of the five basic components of family function (adaptation, partnership, growth, affection and resolve). This test contains 5 items that are presented on a 3-point Likert scale, with responses ranging from 1 “almost always” to 3 “hardly ever”. The total score ranges from 0 to 10. Scores above 7 indicates normal functioning, between 4 and 6 moderate dysfunction, and less than 3 suggests severe dysfunction.

The Rosenberg Self-esteem Scale (Rosenberg, 1979) is a 10-item self-reported scale that examines overall self-worth by measuring people’s positive and negative feelings about themselves. Items are presented on a 4-point Likert scale, with responses ranging from 1 “strongly agree” to 4 “strongly disagree”. Scores over 30 indicate high self-esteem, between 26 and 29 moderate self-esteem, and below 25 low self-esteem.

#### Nutritional and Lifestyle Measures

The Short and Quick tests (Serra Majem et al., 2003) were used to measure lifestyle and compliance with the Mediterranean diet, which is considered physically and nutritionally healthy. The Quick Test is a 16-item self-reported dichotomous questionnaire in which the child’s adherence to the Mediterranean diet is classified as low quality (score below 3 points), regular (score between 4 and 7) or optimal (over 8 points). The Short test contains 2 self-reported items, the number of hours per week that a child spends playing video games or watching TV, and the number of hours doing extracurricular activities. In addition, it allows the child’s lifestyle to be classified as poor (score below 5 in boys, 4 in girls), average (score between 6 and 8 in boys, and 5–7 in girls), and good (score over 9 in boys and 8 in girls).

### e-Health Intervention Components

The intervention consisted of 3 modules targeting nutrition, healthy lifestyle, body concerns and social pressures. An online psychoeducational intervention—with content based on the theory of planned behavior (Ajzen, 1991) to promote healthy nutrition-, lifestyle- and body shape-related behaviors—was delivered to participants who had not been referred at baseline to the primary health care service (see Table 2 for further details on the intervention characteristics).

**Table 2:** Intervention modules and content and behavioral change techniques used in the program

Module	Content
Nutrition	Promoting healthy eating attitudes and behaviors. Following a Mediterranean Diet, analyzing the importance of proteins, carbohydrates, and fats. Promotion of breakfast and regular mealtimes, as well as avoiding sugary beverages, fast food and ready-made food. Dieting, fasting and their risks were also explained in detail
Healthy lifestyle	Raising activity levels, avoiding a sedentary lifestyle, reinforcing the benefits of regular activity for better health, and how activity improves our body satisfaction and how it reflects on self-esteem. Explaining the importance of sleeping enough and the relationship between weight and lack of sleep, as well as the significance of avoiding digital screen time before going to bed
Body concerns and social pressures	Discussing body ideals, the wide range of different body shapes during adolescence, the development of coping skills to address sociocultural pressures to be thin and to critically assess media and societal attitudes about body shape and weight

Determinant of intention (TBP)	Behavior change technique	Application examples
Attitude	Psychoeducation on the behavior- physical/mental health relationship. Positive/negative consequences of behavior (action/inaction)	Requirements of a healthy diet in adolescence and its benefits Dieting and fasting long term consequences and risks Benefits of regular physical activity on body weight regulation, satisfaction and self-esteem. Sleeping and rest patterns in relationship between energy levels and body weight Social media and socio-cultural environment as source of body concern and social pressure to pursue the thin ideal
	Behavioral activation	“You have multiple choices, select what you prefer!”
	Work on the ability to get pleasure through eating healthily, doing regular physical activity, following healthy lifestyle habits and being yourself	The capacity of listening to yourself and encouraging exploration “Cook your own food” “Enjoy new activities”
Subjective norms	Discussion on social pressures to adopt cultural norms (e.g. thin ideal, muscular body in boys…)	Analysing and decoding messages from advertising, media and social media and promotion of critical thinking “What do you think is the main purpose of the advertisement?” “Do you agree or disagree with values underlying the advert and representation of stereotypes?”
Perceived behavioral	Work on self-efficacy and motivation to change	Promote abilities, social support and increased autonomy through tips and instructions to make it easier to achieve goals. (i.e., make changes in your diet one by one to be sure they are sustained in the long term, cook with your family and buy fresh products instead of eating ready made food, increase motivation by doing physical activity with your friends)

*TPB* Theory of Planned Behavior, *Behaviors* the action of eating healthily, doing regular physical activity, sleeping enough, accepting one’s own body, *Intentions* perceived likelihood of eating healthily, doing regular physical activity, sleeping enough, accepting one’s own body

The content was designed by researchers with expertise in Psychology and Nutrition, and was accessible 24/7 via the Moodle platform for 3 months. Each month, a module was progressively unlocked on the intervention students´ platform. At the end of each module, students had a knowledge questionnaire or a practical exercise with predefined feedback answers to encourage changes in behavior (e.g., increasing fruit intake). All of the modules were interactive (e.g., short videos, summary of contents and hands-on activities). The videos were recorded by the lead researcher and focused on nutrition and healthy lifestyles. Additionally, in some cases, selected videos were from recognized organizations in the field (e.g., the Spanish Health Ministry).

At the same time, a 3-session e-Health preventive intervention was delivered by teachers in 55-min lessons. Classroom interventions were solely e-health with teachers playing online videos and materials to further reinforce the main aspects of the students´ intervention platform. To that end, teachers had access to a limited personalized Moodle platform where activity videos, the content to teach in each session, and a short video explaining the intervention protocol were available. Questions or issues were resolved by researchers through email or by phone before each session. The program was entirely online but teachers reinforced various online content in class.

### Primary Outcomes

The primary outcomes were ED risk (EAT-26 > 20) and weight percentiles (underweight = below 3rd, normal weight = between 3rd and 85th, overweight = above 85th and obesity = above 97th percentiles). In addition, changes in self-esteem, family functioning, adherence to the Mediterranean diet and lifestyle were measured. All of the outcome variables were assessed at post intervention.

### Data Analysis

The data were analyzed using SPSS Statistics Software version 23. Preliminary analyses examined the normality of the variables using the Kolmogorov–Smirnov Test. We also inspected inconsistent data as of the responses to the attentional control items, and no data was excluded as a result. Some of the variables were found to not follow a normal distribution whereas others did, hence non-parametric and parametric statistical methods were adopted as appropriate. A complete case analysis and intention-to-treat analysis were performed to evaluate possible differences in main outcome variables. Multiple imputation using the Fully Conditional Specification (FCS) imputation method was performed and five datasets with a maximum of 10 iterations per imputation were generated.

Correlations at baseline were calculated using Spearman correlation tests to assess levels of dependence between the selected variables.

Comparisons between the intervention and control arms in the primary outcome variables were performed using Student´s independent t test (parametric test) for continuous variables, the Mann–Whitney U test (non parametric test) for continuous and ordinal variables depending on the assumption of normality. Binary variables were calculated using the *χ*^2^ test.

Cohen´s effect size formulas for the independent T-test (see Formula 1) and the Mann–Whitney U test (see Formula 2) were used.

1 $$d = \frac{{\left( {M1 - M2} \right)}}{{\sqrt {\frac{{\left( {\left( {n1 - 1} \right)* SD1^{2} + \left( {n2 - 1} \right)* SD2^{2} } \right)}}{{\left( {n1 + n2 - 2} \right)}}} }}$$

2 $$d = Z * \sqrt {\frac{1}{N1} + \frac{1}{N2}}$$

G power Software version 3.1 was used to estimate the statistical power post hoc, with a power level of 80% set as a reasonable balance between alpha and beta risk.

Finally, a moderation analysis was performed using the PROCESS Hayes macro as implemented in SPSS. Specifically, it tested the moderating effects of sex on the relationship between the study group (intervention vs. wait-list) and statistically significant main outcomes. This analysis was conducted on the total sample (161), following an intention-to-treatment approach.

## Results

### Correlations

Statistically significant Spearman correlations between the study variables at baseline are shown in Table 3. There were some small to moderate effect sizes in the total sample and by sex. High statistical power was found in the total sample for BMI/weight and the EAT-26 dieting subscale, weight and physical activity, self-esteem and family function, self-esteem and nutrition, self-esteem and the dieting subscale, physical activity and nutrition, and physical activity and the EAT-26 bulimia subscale.

**Table 3:** Significant correlations between variables

Correlations	Total n = 161 rho	*P*	Boys = 87 rho	*p*	Girls = 74 rho	*p*
BMI-Sub.Dieting	0.417*	0.001	0.471*	0.001	0.357*	0.002
BMI-P.Activity	− 0.180	0.022	− 0.293	0.006		
BMI-EAT-26	0.196	0.001	0.300*	0.005		
Weight-EAT-26	0.201	0.010	0.329*	0.002		
Weight-P.Activity	− 0.228*	0.004	− 0.310*	0.004		
Weight-Sub.Dieting	0.400*	0.001	0.457*	0.001	0.325*	0.005
Weight-Sub.Oral	− 0.182	0.021			− 0.312	0.007
BMI-Sub.Oral	− 0.185	0.001			− 0.240	0.039
RS-Apgar	0.413*	0.001	0.388*	0.001	0.431*	0.001
RS-P.Activity	0.182	0.022	0.239	0.026		
RS-Nutrition	0.239*	0.002	0.277	0.010		
RS-EAT-26	− 0.178	0.024				
RS-Sub.Dieting	− 0.223*	0.004			− 0.255	0.028
RS-Sub.Bulimia	− 0.171	0.030				
P.Activity-Nutrition	0.228*	0.004	0.249	0.021		
P.Activity-Sub.Bulimia	− 0.237*	0.003	− 0.243	0.024		
Sub.Bulimia-Nutrition	− 0.186	0.019				
Sub.Oral-Nutrition	0.179	0.024			0.386*	0.001
Sub.Oral-BMI	− 0.185	0.019			− 0.240	0.039
Sub.Oral-Weight	− 0.182	0.021				

*BMI* Body mass index, *Sub*.*Dieting* Dieting subscale, *P*.*Activity* Short test (Physical Activity), *EAT-26* Eating Attitudes Test, *Sub*.*Oral* Oral control subscale, *RS* Rosenberg Self-esteem Scale, *Apgar* Family Apgar Test, *Nutrition* Quick Test (Nutrition), *Sub*.*Bulimia* Bulimia and food preoccupation subscale

*Power up to 80%

An analysis by sex, in boys, showed that the highest statistical power and largest effect sizes were for BMI and the EAT-26 dieting subscale, BMI and EAT-26, weight and EAT-26, weight and the EAT-26 dieting subscale, weight and physical activity, and self-esteem and family function. In girls, the highest statistical power and largest effect sizes were for the relationship between BMI and the EAT-26 dieting subscale, weight and the dieting subscale, self-esteem and family function, and nutrition and the EAT-26 oral control subscale.

### Intergroup Differences at Post-intervention

#### Complete Case Analysis

Pre-post intervention mean differences and standard deviations in each group are summarized in Table 4.

**Table 4:** Pre-post mean differences in the intervention group (IG) and wait-list control (WLC) in complete case and intention-to-treat analysis

Complete case analysis							Intention-to-treat analysis					
Variable	IG = 59	WLC = 75	U	*p*	d	1 − β	IG = 79^*a*^	WLC = 82^a^	U^b^	*p*^b^	d	1 − β
BMI	− 0.83 (2.39)	− 0.824 (1.82)	1,262.5	0.968			− 0.81 (2.32)	− 0.73 (1.93)	2,285.5	0.846		
EAT26	1.09 (6.62)	− 1.69 (7.14)	1,748.5	0.021*	0.398	0.482	1.08 (6.57)	− 1.43 (7.26)	1,854.5	0.040*	0.323	0.405
Sub.Diet	0.44 (4.38)	− 1.18 (5.13)	1,987.0	0.138			0.45 (4.35)	− 1.18 (5.09)	1,987.0	0.138		
Sub.Bulimia	− 0.27 (2.39)	− 0.27 (2.16)	2,208.5	0.542			− 0.27 (2.37)	− 0.27 (2.15)	2,452.5	0.542		
Sub.Oral	0.92 (2.64)	0.06 (3.17)	1,853.0	0.038*	0.359	0.489	0.92 (2.62)	0.06 (3.15)	1,853.0	0.038*	0.327	0.403
RS	2.61 (5.63)	2.83 (6.17)	2,121.0	0.838			2.67 (5.59)	2.77 (6.12)	2,433.0	0.659		
Apgar	1.07 (1.93)	0.96 (2.56)	1,921.0	0.333			1.11 (1.93)	0.92 (2.55)	2,095.0	0.304		
P.Activity	0.61 (2.32)	1.25 (2.27)	1,821.5	0.076			0.61 (2.30)	1.20 (2.27)	2,700.5	0.108		
Nutrition	0.58 (3.20)	0.01 (2.94)	2,002.5	0.455			0.63 (3.19)	0.01 (2.89)	2,104.5	0.328		

*IG* Intervention group, *WLC* Wait-list control, *EAT-26* Eating Attitudes Test, *Sub*.*Dieting* Dieting subscale, *Sub*.*Bulimia* Bulimia and food preoccupation subscale, *Sub*.*Oral* Oral control subscale, *RS* Rosenberg Self-esteem Scale, *Apgar* Family Apgar Test, *P*.*Activity* Short Test (Physical Activity), *Nutrition* Quick Test (Nutrition)

*Statistically significant *p* < 0.05

^a^Five imputations pooled data

^b^Data from number 5 imputation, significant results were similar between imputations

The Mann–Whitney U test showed differences in the pre-post scores of the EAT-26 and the EAT-26 oral control subscale between the complete cases of IG and WLC groups. Specifically, the IG group had significantly greater reductions in scores in the EAT-26 (*Mpost-treatment* =  − 2.773) and oral control subscale (*Mpost-treatment* =  − 0.852) to a greater extent than the WLC. The Mann–Whitney U test and Student´s independent t test did not show significant differences by sex between groups in the variables studied (*p* = from 0.078 to 0.992).

Finally, there were 15 participants above the cut-off point of 20 (ED risk) according to the EAT-26. Twelve of those were in the WLC and 3 in the IG. Nonetheless, there were no significant differences from a statistical point of view [*χ*^2^(1,*N* = 134) = 3.753; *p* = 0.053].

#### Intention-to-Treat Analysis

The Mann–Whitney U test showed differences between the IG and WLC groups in the pre-post scores of the EAT-26, and the EAT-26 oral control subscale.

The IG exhibited greater reduction than the WLC in the EAT-26 (*Mpost-treatment* =  − 2.515) and oral control subscale (*Mpost-treatment* =  − 0.852). The Mann–Whitney U test did not show significant differences by sex between groups (*p* = from 0.050 to 0.964) in the variables studied.

### Moderation Analysis

There were no statistically significant moderation effects of sex in the outcome variables that exhibited statistically significant differences (see Table 5).

**Table 5:** Moderation analysis of the correlated variables

Independent variable (X): allocation (IG or WLC)					
Sample	Dependent variable (Y)	Moderator variable (M)	β	C.I. 95%	*p*
Both sexes n = 161	EAT-26 pre-post	Sex	− 1.555	− 3.404, 0.294	0.099
Both sexes n = 161	Sub.oral pre-post	Sex	− 0.104	− 0.922, 0.715	0.804

*IG* Intervention group, *WLC* Wait-list control, *EAT-26* Eating Attitudes Test, *Sub*.*Oral* Oral control subscale

## Discussion

This study provided evidence of the short-term effectiveness of a psychoeducational e-Health intervention for reducing ED risk. Compared to a wait-list condition, the e-Health prevention program led to a statistically significant reduction in oral control subscale behaviors post-intervention. There were no significant sex differences. Notably, we did not find evidence for most of the EAT-26 subscales, suggesting that the reduction in ED risk may be primarily attributable to the impact of the program on self-control of eating (e.g., eating restricted behaviors).

In this study, correlational analyses showed a relationship between baseline BMI, weight (in the total sample and by sex), self-esteem (in the total sample, but not by sex) and the EAT-26 dieting subscale. Based on these results and prior research, it may be that individuals with higher BMIs, greater weight, and low self-esteem have more concerns about shape and weight and could be more likely to be at risk of developing EDs (Puhl & Lessard, 2020; Uchôa et al., 2019).

Prior studies have shown that selective prevention e-Health interventions are associated with significant decreases in body and weight concerns, dietary restriction, drive for thinness, internalization of the thin ideal and symptoms of EDs at post-intervention (Harrer et al., 2019; Linardon et al., 2020). Similarly, universal prevention programs developed for adolescents based on media literacy have produced positive results in body and weight concerns (Kurz et al., 2021; Le et al., 2017). In this study, the post-intervention findings showed that there were no differences in BMI between the intervention and control arms, but the IG group had lower scores in the EAT-26 and oral control subscale. The findings are in accordance with research showing that digital interventions which target central maintaining factors produce significant changes in core ED symptoms (Linardon et al., 2021). More generally, the effectiveness in this study may be related to the content delivered in the experimental group in the intervention, which goes far beyond solely informational content (Burkhart et al., 2022). The e-Health intervention addressed nutritional aspects (healthy nutrition and information on dieting risk and consequences) along with body concerns and social pressures (training in coping skills to address sociocultural pressures to be thin and to critically assess media and societal attitudes about body shape and weight) usually included in most successful prevention programs factors (dietary restraint, drive for thinness, and thin-ideal internalization, among others) (Ciao et al., 2014).

In addition, the program aimed to educate participants specifically about binge eating, its causes and consequences, and how to prevent it (e.g., eating 5 meals a day). There is also a link between attitude and behavior (Ajzen, 1991), and the findings suggest that the intervention might have arguably produced a reduction in the intention to lose weight through decreases in concerns about food, body weight or problematic eating behaviors. The blended delivery mode (in person, during classroom time, and virtually at home) may also have contributed to the beneficial effects. Self-guided Internet-based interventions for ED symptoms usually produce suboptimal rates of adherence and retention, and hence small intervention effects (Linardon et al., 2022). In contrast, interventions delivered by teachers have been shown to produce significant changes in nutrition and dietary habits (Cotton et al., 2020). In this study, the implementation of the program during classroom hours, delivered by teachers, may have increased participants’ motivation to engage and interact with materials. Teachers were also supported by the research staff, and were supervised weekly to ensure that they faithfully implemented the preventive program.

On a related note, the program was online and so participants would be expected to have had effective exposure to the program. The content was accessible 24/7 and given that a digital device was required to access the program, most families were expected to be involved. Although it was not evaluated, it is arguable that families helped the young people to interpret messages and exercises and that may have fostered critical thinking in families as well. The literature has previously reported that interventions involving parents can boost the effectiveness of school-based prevention programs (Cuijpers, 2002). In our program, families were informed beforehand about the characteristics of the intervention, and parents may well have been interested and present whenever children accessed the content. In this vein, parents might have helped to reduce ED risk behaviors through increased parent–child communication and enhanced monitoring of children’s behaviors. Unfortunately, due to time constraints in participants’ schedules, we were unable to consider these variables as potential moderators of effectiveness, and further research is warranted to examine this important aspect more thoroughly.

Another likely explanation of the current findings is related to the inclusion of behavioral activation, which has been shown to be successful in improving diet quality in adults (Paans et al., 2020). This technique aimed to change food-related behaviors and maladaptive eating patterns to improve diet. It also sought to increase participants’ engagement with the Mediterranean diet through promotion of exercise and new, rewarding recipes.

The study is not without limitations. Firstly, the results may be biased because of the relatively small sample size. Secondly, because of the pandemic, face-to-face follow-up assessments were not feasible as planned and long-term effects of the e-Health intervention cannot be concluded. On a related note, the COVID-19 pandemic may have influenced family function and self-esteem, and may have biased the results. Furthermore, in part due to time constraints during teaching hours, other potentially relevant outcome variables were not explored. Similarly, it was not possible to perform a process evaluation (e.g., adherence of the teachers to the program curriculum). On another note, feasibility and accessibility are two variables that should be considered as well, particularly to gather information on teachers’ and families’ acceptance levels and barriers towards accessing and adhering to the content. Future studies should gather data on these aspects as well as on potential moderators and mediators framed by TPB. The RE-AIM evaluation framework is a suitable and widely accepted approach for this endeavor that suggests using a 6 month-2 + year assessment time frame (Glasgow et al., 1999). Lastly, given the number of multiple tests, type I error might be operating here. No adjustments were made for multiple comparisons and conclusions should be tempered in this regard. Additionally, future research should include a higher number of data points to report on long-term efficacy and ensure sufficient power to conduct longitudinal analyses (preferably mixed ANOVA or Generalized Estimated Equations (GEE).

## Conclusions

Taken together, the results from the present study suggest the advisability of promoting critical thinking on beauty ideals, healthy nutrition and weight satisfaction, which is considered a protective factor against intention to diet (Nejad et al., 2004). The implementation of universal e-Health interventions including tutor-led digital components seems to be effective in reducing the risks of ED in children. Given that EDs occur early in life (13 years old), schools should advocate and promote the implementation of universal interventions. Online delivery of the program has several advantages over traditional in-person programs. It may be expected to be cost-effective as it could decrease the burden on the healthcare system and save overall costs, which may enhance sustainability. In addition, teachers could act as facilitators and encourage parental involvement to monitor and detect ED early. Though the results are encouraging, further replication with larger sample sizes and long-term follow-ups are needed. In this regard, future studies should conduct both medium (e.g., 3–6 months) and long-term (e.g., one year) follow-up assessments.
